# Structures and activities of Drug and Therapeutic Committees in formulary management: findings from a national survey and key informant interviews

**DOI:** 10.1186/1471-2458-14-S1-O10

**Published:** 2014-01-29

**Authors:** Azuana Ramli, Syed Mohamed Aljunid, Saperi Sulong, Faridah Aryani Md Yusof

**Affiliations:** 1 United Nations University International Institute for Global Health (UNU-IIGH), 56000 Cheras, Kuala Lumpur, Malaysia; 2 Pharmaceutical Services Division, Ministry of Health, Kuala Lumpur, Selangor 46790, Malaysia; 3 Universiti Kebangsaan Malaysia, Jalan Yaacob Latif, Bandar Tun Razak, 56000 Cheras, Kuala Lumpur, Malaysia

## Background

Drug expenditures continue to rise due to increasing disease burden and emergence of new drugs. World Health Organization (WHO) recommended that Drug and Therapeutic Committee (DTC) be set up in healthcare institutions to manage drug formularies and policies promoting rational drug selection and use. Operations of DTCs in MOH facilities are yet to be documented. This study was set to gain insights on DTCs particularly in exerting these roles.

## Materials and methods

A mixed method of focus group discussions (FGD), survey and semi-structured interviews was used. The FGDs provided basic information on DTC operations and formulary management in MOH facilities. Survey questionnaires were sent to all 136 MOH hospitals and 15 state health offices to obtain more detailed information. Seven key informants were interviewed to gain in-depth knowledge.

## Results

Altogether 118 completed questionnaires were returned (response rate: 78%). Almost all (97.5%) of the facilities had operational DTCs. However, they varied considerably in structures and activities. All DTCs were multidisciplinary consisting of mainly department heads with 3 to 36 members. There were 96 of 118 (81.4%) facilities who maintained local drug list that are subsets to the National Drug Formulary. Eighty-one (68.6%) of the DTCs lacked term of reference documents. Expected DTCs’ responsibilities were not clearly specified, often being assumed by pharmacy departments. The interviews highlighted that local DTCs’ fundamental agenda was managing individual institutions’ drug expenditures, supporting the survey findings that drug budget and procurements being the two most frequently discussed issues in DTC meetings. New applications to list new drugs into the national formulary often not debated much during local DTC meetings. The main reasons identified include inadequate resources to conduct drug evaluation and perceptions of overlapping roles with national level DTCs.

## Conclusions

Suboptimal standardisation in structure and activities of MOH DTCs emphasises the need for national level coordination of individual DTC operations in formulary management roles.

